# Profiling anti-cyclic citrullinated peptide antibodies in patients with juvenile idiopathic arthritis

**DOI:** 10.1186/1546-0096-10-29

**Published:** 2012-08-29

**Authors:** Anne E Tebo, Troy Jaskowski, K Wayne Davis, April Whiting, Bronte Clifford, Andrew Zeft, Bernadette McNally, Harry R Hill, John Bohnsack, Sampath Prahalad

**Affiliations:** 1 Associated Regional and University Pathologists Institute for Clinical and Experimental Pathology, Salt Lake City, UT, USA; 2 Department of Pathology, University of Utah, Salt Lake City, UT, USA; 3 Department of Pediatrics, University of Utah, Salt Lake City, UT, USA; 4 Department of Medicine, University of Utah, Salt Lake City, UT, USA; 5 Department of Pediatrics and Human Genetics, Emory University School of Medicine, 2015 Uppergate Drive NE, Atlanta, GA, 30322, USA; 6 Children's Healthcare of Atlanta, Atlanta, GA, USA

**Keywords:** Juvenile idiopathic arthritis, Serologic markers, Cyclic citrullinated peptide, Rheumatoid arthritis

## Abstract

**Background:**

Anti-citrullinated protein/peptide antibodies (ACPA), have high specificity for rheumatoid arthritis (RA). Some children with juvenile idiopathic arthritis (JIA), phenotypically resemble RA and test positive for rheumatoid factor (RF) a characteristic biomarker of RA. We investigated the prevalence of ACPA and its relationship to other serologic markers associated with RA in a well-characterized JIA cohort.

**Methods:**

Cases were 334 children with JIA, 30 of whom had RF + polyarticular JIA. Sera from all cases and 50 healthy pediatric controls were investigated by ELISA at a single time point for anti-cyclic citrullinated peptide (anti-CCP) IgG, RF IgM, IgA and IgG, anti-RA33 IgG, and antinuclear antibodies (ANA). Comparisons between cases and controls were made using Chi-square or Fisher exact tests and T-tests.

**Results:**

The prevalence of RF was 8% among controls, and 12% among cases (ns). The prevalence of ACPA was 2% in controls and 14.3% in cases (OR 8.2, p <0.01). Children who were ACPA-positive and RF-negative (n = 23) had a significantly earlier onset-age (4.6 years vs. 12.1 years, p <0.00001) and had fewer HLA-DRB1 shared epitope alleles than those positive for both RF and ACPA (n = 25). Prevalence of anti-RA33 was not different between cases and controls.

**Conclusions:**

ACPAs are detectable in 14% of children with JIA. Children with positive ACPA but negative RF are frequent, and may define a distinct subset of children with JIA. ACPA testing should be included in the classification of JIA.

## Background

Juvenile idiopathic arthritis (JIA), the most common cause of chronic arthritis in children, is a heterogeneous group of chronic arthropathies with different clinical, genetic and serological profiles [1]. One subtype of JIA, characterized by rheumatoid factor (RF) positive polyarthritis, phenotypically resembles rheumatoid arthritis (RA) in adults, and seems to represent the childhood onset of RA. A number of clinical studies have shown that sera from patients with JIA may contain other serologic markers such as, anti-perinuclear factor (APF), anti-keratin (AKA), anti-cyclic citrullinated peptide (CCP) and anti-RA33 antibodies typically associated with RA [2-4]. Recent studies have demonstrated anti-citrullinated protein/peptide antibodies (ACPA) which include anti-CCP antibodies, have high specifity for RA, and are now included in the revised diagnostic criteria for RA [5]. Furthermore, there is evidence that ACPA-positive and ACPA-negative RA patients display significant risk allele frequency differences which are mainly confined to the HLA region [6].

Prior investigations of the prevalence of ACPA in children have mostly been in small cohorts of children with JIA, with very few cases of RF-positive polyarticular JIA included [2,7-20]. Although the overall prevalence of ACPA in JIA in these studies was low, a substantial proportion of RF-positive patients with the polyarticular subtype have these antibodies [11,13,15,19]. We sought to investigate the prevalence of ACPA in a large well-characterized cohort of JIA and investigate the relationship between these antibodies with other serologic markers for RA.

## Methods

Cases were 334 children (65.5% female) diagnosed with JIA using the International League of Associations for Rheumatology (ILAR) criteria [1]. All subjects had been followed at the Pediatric Rheumatology outpatient clinics at Primary Children’s Hospital, Salt Lake City, UT, USA. The mean age of onset in the JIA cohort was 6.7 years. Controls were 50 healthy children (50% female) with no current or previous history of JIA recruited at ARUP Laboratories, Salt Lake City, UT, USA. Controls had a mean age of 11.5 at the time of blood draw. The study was approved by the Institution Institutional review Board at University of Utah, Salt Lake City, UT, USA, and all subjects gave informed consent for the study.

Serum was separated soon after blood was drawn and samples were stored at −80°C until investigated. Sera from all JIA patients and controls were investigated at a single time point for all analytes. The assays were performed and evaluated by operators who were blinded to other serological results and unaware of the patients’ clinical data. ACPA were tested by ELISA (INOVA Diagnostics, San Diego, CA, USA; IgG/A anti-CCP3). Positive ACPA was defined as a serum concentration >20 Units. IgM, IgA and IgG RF were detected and quantified by ELISA as recommended by the manufacturer (Theratest, Lombard, IL, USA), with values greater than 26, 36 and 21 units respectively defined as positive. Anti-RA33 IgG antibodies were detected and semi-quantified by ELISA as recommended by the manufacturer (IMTEC, Wiesbaden, Germany); ≥25 Units were considered positive. Antinuclear antibodies (ANA) were initially screened by ELISA (INOVA Diagnostics, San Diego, CA, USA) and positive results confirmed by indirect immunofluorescence assay (IFA) on HEp-2 cells at a dilution of 1:40 (INOVA Diagnostics, San Diego, CA, USA). High-resolution HLA-DRB1 genotyping was performed on 21 Non-Hispanic White cases positive for IgM-RF and ACPA, as well as 22 Non-Hispanic White cases positive for ACPA but negative for IgM-RF. Comparisons between cases and controls were made using Chi-square or Fisher exact tests for categorical variable and T-tests for continuous variables.

## Results

The distribution of the different JIA subtypes and prevalence of the specific antibodies measured is shown in Table 1. Of the cases, 31 (9.2%) had systemic JIA, 30 (9.0%) had RF-positive polyarticular JIA, 76 (22.7%) had RF-negative polyarticular JIA, 119 (35.6%) had persistent oligoarticular JIA, 26 (7.8%) had extended oligoarticular JIA, 27 (8.1%) had enthesitis related arthritis (ERA) and 25 (7.5%) had undifferentiated JIA. The prevalence of RF antibody isotypes ranged from 0-8% among controls, and 8-12% among all JIA cases (Table 2). As anticipated, children classified as having RF + polyarticular JIA per ILAR criteria had significantly greater prevalence of RF-IgM, RF-IgG, and RF-IgA, compared to controls. The prevalence of RF-IgM was 93% in this group, reflecting that some who tested positive in the past were negative at the time of this study. RF-IgM was also positive in a minority of subjects with other JIA subtypes who had previously tested negative for RF-IgM at the time of diagnosis (Table 1). RF-IgA and RF-IgG were also detected in some cases with JIA.

**Table 1:** Characteristics of the cohort and prevalence of the biomarkers in the different JIA subtypes

**Variable**	**All JIA N = 334**	**Systemic N = 31**	**RF + polyarticular N = 30**	**RF negative polyarticular N = 76**	**Persistent oligoarticular N = 119**	**Extended oligoarticular N = 26**	**Enthesitis related arthritis N = 27**	**Undifferentiated arthritis N = 25**
Onset Age*	6.5	6.1	11.2	6.6	5.7	3.5	9.6	7.7
Females	65	45	97	75	63	81	33	72
CCP IgG	14	13	73	8	4	19	4	20
RF IgM	12	3	93	1	2	0	4	24
RF IgG	8	0	43	4	6	4	7	0
RF IgA	8	6	70	0	1	0	4	8
Anti RA33 IgG	6	3	3	16	3	12	0	4
ANA IFA	26	3	17	24	41	35	7	16

* All values are percentages, except onset age given in years.

**Table 2:** RF positive poly and all JIA vs controls

	**Controls**		**All JIA**				**RF + poly JIA**			
	**N (%)**	**Titer mean U**	**N (%)**	**Titer mean U**	**OR (95 % CI)**	**P**	**N (%)**	**Titer mean U**	**OR (95 % CI)**	**P**
ACPA (CCP IgG)	1 (2)	8	48 (14)	25	8.22 (1.2-163.9)	0.01	22 (73)	295	135 (15–3085)	<0.00001
RF IgM	4 (8)	10	39 (12)	19	1.52 (0.49-5.27)	ns	28 (93)	118	161 (23–1542)	<0.00001
RF IgG	4 (8)	10	26 (8)	12	0.97 (0.30-3.45)	ns	13 (43)	30	8.79 (2.23-37.7)	0.0002
RF IgA	0 (0)	6	27 (8)	22	-	0.03	21 (70)	140	-	<0.00001
Anti RA33	3 (6)	18	21 (6)	14	1.05 (0.28-4.61)	ns	1 (3)	15	0.54 (0.02-6.31)	ns
ANA IFA	3 (6)	-	88 (26)	-	5.60 (1.62-23.16)	0.002	5 (17)	-	3.00 (0.56-17.59)	ns

Controls = 50, cases = 334, and RF positive poly JIA cases 30. Means of ACPA, RF, RA33 values presented as Units/dL Numbers of subjects positive for ANA by immunoflorescence at titers ≥ 1:40 are indicated.

ACPA were detected in one control and 48 cases (2% vs. 14.3%; OR 8.2, p <0.01). In all, 22 of 30 (73%) children previously classified as RF + polyarticular JIA were positive for ACPA. Twenty-five JIA cases were positive for both RF-IgM and ACPA, and generally had higher titers of ACPA than those who were positive only for ACPA (median titer of 358 vs. 27), (Table 3). Of particular interest were 23 children who had a positive ACPA and were negative for RF (IgM, IgG or IgA). Phenotypic characteristics of this group of children were compared to 25 children who were positive for both RF and ACPA (Table 4). Children who were ACPA-positive and RF-negative had a significantly earlier age of onset (4.6 years vs. 12.1 years, p <0.00001 by T-test), included more males (8/23 vs. 2/25, p <0.01), and included children with polyarticular (n = 6) as well as oligoarticular onset (n = 9). High resolution *HLA-DRB1* genotyping indicated that Non-Hispanic White cases who were RF-/ACPA + had fewer shared epitope alleles compared to those who were RF+/ACPA + (9/21 vs. 18/21, OR 0.12 (0.02-0.61), p <0.002). A notable finding was that three children that were ACPA + and had 2 positive RF-IgM tests were classified as “undifferentiated JIA” per the ILAR criteria: two girls had oligoarticular onset, and one boy with onset after the age of 6 years was positive for HLA-B27. All three children carried HLA-DRB1 alleles encoding the shared epitope.

**Table 3:** RF/ACPA proportions and characteristics

	**Controls**			**Cases**					
	**N (%)**	**CCP titer**	**RF titer**	**N (%)**	**CCP titer**	**RF titer**	**Onset Age**	**Joints at onset**	**ESR at onset**
RF IgM – ACPA IgG -	45 (90)	3.9	6.5	272 (81.4)	0	6	5.3	2	19
RF IgM + ACPA IgG -	4 (8)	2.5	31	14 (4.2)	0	47.5	8.0	4.5	28
RF IgM – ACPA IgG +	1 (2)	185	0	23 (6.9)	27	5	4.6	3	26
RF IgM + ACPA IgG +	0 (0)	-	-	25 (7.5)	358	137	11.5	8	22

There were 50 controls and 334 cases. Titers refer to median values except with reference to RF-/CCP + control where it refers to the actual value. Onset age, joints at onset and ESR at onset are median values.

**Table 4:** Phenotypic features of children who are ACPA and RF positive versus those ACPA positive and RF negative

**Feature**	**RF IgM-/ACPA + (n = 23)**	**RFIgM+/ACPA + (n = 25)**	**P value**
Males, N	8	2 (8 %)	<0.03
Onset age, years	4.6	12.1	<0.0001
Mean RF IgM titer, units	6	129	<0.0001
Mean RF IgG titer, units	7	34	0.007
Mean RF IgA titer, units	6	161	<0.0001
Mean ACPA titer, units	33	282	<0.0001
HLA-DR SE positive N (%)	9/21 (43%)	18/21 (86%)	<0.003
ILAR Subtypes			
Persistent oligoarthritis	4	0	-
Extended oligoarthritis	5	0	-
RF - polyarthritis	6	0	-
RF + polyarthritis	0	22	-
ERA	1	0	-
Systemic JIA	4	0	-
Undifferentiated JIA	2	3	-

SE; As previously reported, *HLA DRB1 0101, 0102, 0401, 0404, 0405, 0408, 1001, 1302* were designated at SE alleles.

Individually, both RF-IgM and ACPA had poor sensitivity for JIA as a whole, but had high specificity (Table 5). However, for RF + polyarticular JIA, while ACPA had a lower sensitivity compared to RF (0.73 for ACPA and 0.93 for RF), ACPA had better specificity and positive predictive values (0.98 and 0.98 versus 0.92 and 0.88 respectively).

**Table 5:** Sensitivity, specificity, positive and negative predictive values of CCP and/or RF

	**All JIA**				**Polyarticular RF-positive JIA**			
	**Sensitivity**	**Specificity**	**PPV**	**NPV**	**Sensitivity**	**Specificity**	**PPV**	**NPV**
ACPA IgG	0.14	0.98	0.98	0.15	0.73	0.98	0.98	0.86
RF-IgM	0.12	0.92	0.91	0.13	0.93	0.92	0.88	0.96
ACPA IgG and RF IgM	0.07	1.00	1.00	0.14	0.73	1.00	1.00	0.86
ACPA IgG or RF IgM	0.19	0.90	0.95	0.14	0.93	0.90	0.85	0.96

ACPA: anti citrullinated peptide antibody (CCP IgG), RF: rheumatoid factor. PPV: positive predictive value; NPV- negative predictive value.

To determine the prevalence of ANA antibodies in our JIA cohort compared to healthy controls, all samples were screened with an ANA ELISA and the presence of a specific pattern and titer confirmed by IFA (Table 1). Only 3 controls had titers of ≥ 1:40 compared to 96 cases [6% vs. 26%, OR 5.6 (1.6-23.2), p <0.01]. The prevalence of RA-33 antibodies was not significantly different between cases and controls, (6% each). The prevalence of RA-33 antibodies among different JIA subtypes was also not different compared to controls. There was no difference in the mean titers of RA33 antibodies between cases and controls.

There have been several prior investigations into the prevalence of ACPA in children with JIA with variable results (Table 6). These studies had a median of 78 subjects with JIA (range 45–230). Moreover, these studies had very few children with the RF-positive polyarticular subtype of JIA, median 11 (range 1–20). The prevalence of ACPA in these studies varied from 1.8% to 77.3%. The prevalence of ACPA in RF-positive poly JIA varied from 0-100% in these studies. We calculated the prevalence of ACPA in children with non-RF-positive polyarticular JIA in these studies and found the mean prevalence of ACPA to be 6%, similar to the 6.9% observed in our study.

**Table 6:** prior studies of ACPA in JIA

**Author (Ref)**	**Year**	**All JIA**		**RF-positive polyarticular JIA**		**Controls**	
		**N**	**Prevalence of ACPA**	**N**	**Prevalence of ACPA**	**N**	**Prevalence of ACPA**
Avcin [2]	2002	109	1.8	1	0.0	30	0.0
Hromadnikova [13]	2002	140	5.0	18	11.1	24	0.0
Van Rossum [20]	2003	71	14.1	11	72.7	-	-
Low [17]	2004	66	77.3	16	75.0	25	0.0
Kasapcopur [14]	2004	122	2.5	12	25.0	15	0.0
Kwok [16]	2005	59	10.2	5	80.0	60	0.0
Ferucci [9]	2005	230	5.7	14	57.1	688	0.6
Brunner [8]	2006	45	4.4	2	100.0	42	0.0
Kuna [15]	2009	56	1.8	2	50.0	17	0.0
Habib [12]	2008	68	20.6	20	70.0	20	0.0
Gupta [11]	2010	78	23.1	8	87.5	0	-
Morbach [21]	2010	191	2.6	6	66.7	88	1.1
Gilliam [10]	2011	96	14.6	16	56.3	10	-
Present study	2012	334	14	30	73.3	50	2.0
Total		**1665**	**14.1**	**161**	**58.9**	**1069**	**0.33**

Prior published studies that investigated prevalence of ACPA in JIA. First author, year of publication and reference number are indicated. The mean prevalence is provided in the last row for all JIA, polyarticular RF-positive JIA and controls. After excluding subjects with RF-positive polyarticular JIA, the prevalence of ACPA is 6% in JIA.

## Discussion

ACPA characterize the immune response to citrullinated peptide antigens that are a hallmark of seropositive adult RA. ACPA can precede the development of RA by several years and also demonstrate a high specificity for RA [21]. The current generation of ACPA test kits also approach the sensitivity of RF, which generally have a lower specificity for RA. This has resulted in the incorporation of the ACPA status in clinical practice. Additionally, ACPA identify a distinct subset of RA highlighted by several genetic association studies [22]. Smoking has been implicated to be involved in triggering the process of citrullination that eventually leads to auto-immune activation in susceptible hosts [23]. Other factors involved in citrullination include periodontal disease and infections [24-27]. It is likely that the mechanism of citrullination is different in children than in adults, with infections playing a greater role than smoking. In a recent study, Gilliam et al., suggested that in children with IgM-RF positive polyarticular JIA, fibrinogen is the target of citrullination [9].

Our analysis of published studies indicate that in children with JIA who do not have the RF-positive polyarticular subtype, the prevalence of ACPA is ~6%. In our study we observed a significant number of children who were negative for RF-IgM but positive for ACPA. These children phenotypically differed from those who were positive for both RF-IgM and ACPA and are less likely to carry shared epitope encoding HLA DRB1. It should be noted that most of the 23 children who were RF-IgM negative and ACPA positive had low titers of ACPA (range 21–75, median 27). These observations suggest that a low titer of ACPA in the absence of RF-IgM, identifies a subset of JIA that is different from the RF-positive polyarticular JIA subset. Lack of follow -up-studies to determine if these children subsequently developed RF-IgM is a limitation of our study. We observed that children who were positive for both RF-IgM and ACPA phenotypically resemble adults with RA, and also encompass the typical child with polyarticular “RF positive JIA.” We propose that in children who are RF-IgM negative, a higher threshold for ACPA-positivity might be prudent in order to identify those children who have a phenotype compatible with RA. It should be noted that higher titers of ACPA are given more weight in the revised criteria for RA [5].

Our study also highlights some limitations of the ILAR classification criteria. For instance, we identified 3 children who phenotypically resembled seropositive RA, (two positive tests for RF-IgM, and positive for ACPA, carriage of the HLA DRB1 shared epitope) but had to be classified as “undifferentiated JIA” due to the specific requirements of the ILAR criteria. Future revisions of ILAR criteria should take into consideration inclusion of children with fewer than 5 joints who otherwise have biomarkers such as RF-IgM/ACPA, as well as to limit some of the exclusion criteria such as family history of psoriasis or positive HLA B27, if both RF-IgM and ACPA are positive. Presence of both these biomarkers clearly identifies a phenotype akin to adult seropositive RA.

Although our study included a relatively large cohort of children with JIA tested for ACPA, it did have some limitations. A larger cohort of healthy control children would have been ideal. However, the frequency of ACPA in our controls was comparable to other published cohorts. Several of the cases with only positive ACPA had low titers. Our study was also cross-sectional and, therefore, did not address the relationship between the various biomarkers and treatment, disease activity and long term outcome. We have shown that children with RF/ACPA-positive JIA demonstrate similar HLA-DRB1 genotypes as adults with RA [28]. In the present study, the objective was to investigate if children who were ACPA+/RF- differed compared to ACPA+/RF+. Therefore, we limited HLA typing to RF+/ACPA+ and RF-/ACPA + subjects.

Another notable finding from this study is that anti-RA33 antibodies do not appear to be associated with the JIA phenotype. Anti-RA33 antibodies are directed against a nuclear protein antigen identical to the A2 protein of the heterogeneous nuclear ribonucleoprotein. Anti-RA33 antibodies were first described to be prevalent in sera from adults with RA [29]. There have been three prior studies of RA33 in small JIA cohorts. Gabay et al., reported that anti-RA33 antibodies were detected in 11% of sera from 124 children with JIA, and none of the controls [3]. Children with RF + polyarticular JIA had a higher prevalence of antri-RA33 antibodies (57%), although only 7 children with RF + polyarticular JIA were included in this analysis. Nesher et al., also reported finding RA33 antibodies in sera from children with polyarticular as well as oligoarticular JIA [30]. In a recent study of 42 Egyptian patients with JIA, anti-RA33 antibodies were detected in 83% of JIA patients with polyarticular onset, 33% with oligoarticular onset and 57% with systemic onset disease according to a study-specific cut off [31]. By contrast in our study, the prevalence of anti-RA33 antibodies was low and was not significantly different between cases and controls. We also did not observe a higher prevalence of this specific antibody maker namely; among those with polyarticular JIA. The reasons for the different results observed could be due to a combination of factors, different methodologies cutoffs, sample sizes, composition of JIA subtypes, effects of storage, as well as true biologic differences.

Children with positive ACPA but negative RF are frequent and may define a distinct subset of children with JIA. If validated in other large JIA cohorts, ACPA testing should be included in the classification of JIA.

## Competing interests

The authors declare that they have no competing interests.

## Authors’ contributions

AET: Participated in the design of the study, directed the assays used in the study and co-wrote the manuscript. TJ tracked and organized data during the study,and performed the assays used in the study, KWD performed the assays and helped to draft the manuscript. AW: Participated in the design of the study, sample acquisition, and drafting of the manuscript. BC: Enrolled subjects, involved in acquisition of samples and assisted with data collection. ASZ: Participated in the design of the study, and helped to draft the manuscript. BMcN: Participated in the design of the study, helped with data collection and helped to draft the manuscript. HRH: Participated in the design of the study, and helped to draft the manuscript. JFB: Participated in the design of the study, and helped to draft the manuscript. SP: Senior author; conceived of the study, participated in the design and coordination of study, performed the statistical analysis, and wrote the manuscript. All authors participated in the writing, read and approved the final version of manuscript.

Supported by, The National Institute of Arthritis and Musculoskeletal and Skin Diseases (R01-AR060893), The Arthritis Foundation, and The Val A Browning Charitable Foundation, Salt Lake City, UT, The ARUP Institute of Clinical and Experimental Pathology, Salt Lake City., and the Marcus Foundation Inc, Atlanta, GA.

## References

[B1] Petty RE Southwood TR Manners P Baum J Glass DN Goldenberg J International League of Associations for Rheumatology classification of juvenile idiopathic arthritis: second revision, Edmonton, 2001 J Rheumatol 2004 31 390 392 14760812

[B2] Avcin T Cimaz R Falcini F Zulian F Martini G Simonini G Prevalence and clinical significance of anti-cyclic citrullinated peptide antibodies in juvenile idiopathic arthritis Ann Rheum Dis 2002 61 608 611 10.1136/ard.61.7.608 12079901 PMC1754144

[B3] Gabay C Prieur AM Meyer O Occurrence of antiperinuclear, antikeratin, and anti-RA 33 antibodies in juvenile chronic arthritis Ann Rheum Dis 1993 52 785 789 10.1136/ard.52.11.785 7504436 PMC1005189

[B4] Nesher G Moore TL Grisanti MW el-Najdawi E Osborn TG Antiperinuclear factor in juvenile rheumatoid arthritis Ann Rheum Dis 1992 51 350 352 10.1136/ard.51.3.350 1575580 PMC1004659

[B5] Aletaha D Neogi T Silman AJ Funovits J Felson DT Bingham CO 3rd 2010 Rheumatoid arthritis classification criteria: an American College of Rheumatology/European League Against Rheumatism collaborative initiative Arthritis Rheum 2010 62 2569 2581 10.1002/art.27584 20872595

[B6] Padyukov L Seielstad M Ong RT Ding B Ronnelid J Seddighzadeh M A genome-wide association study suggests contrasting associations in ACPA-positive versus ACPA-negative rheumatoid arthritis Ann Rheum Dis 2011 70 259 265 10.1136/ard.2009.126821 21156761 PMC3015094

[B7] Brunner J Sitzmann FC The diagnostic value of anti-cyclic citrullinated peptide (CCP) antibodies in children with Juvenile Idiopathic Arthritis Clin Exp Rheumatol 2006 24 449 451 16956438

[B8] Ferucci ED Majka DS Parrish LA Moroldo MB Ryan M Passo M Antibodies against cyclic citrullinated peptide are associated with HLA-DR4 in simplex and multiplex polyarticular-onset juvenile rheumatoid arthritis Arthritis Rheum 2005 52 239 246 10.1002/art.20773 15641089

[B9] Gilliam BE Reed MR Chauhan AK Dehlendorf AB Moore TL Evidence of fibrinogen as a target of citrullination in IgM rheumatoid factor-positive polyarticular juvenile idiopathic arthritis Pediatr Rheumatol Online J 2011 9 8 10.1186/1546-0096-9-8 21439056 PMC3071779

[B10] Gupta R Thabah MM Vaidya B Gupta S Lodha R Kabra SK Anti-cyclic citrullinated peptide antibodies in juvenile idiopathic arthritis Indian J Pediatr 2010 77 41 44 10.1007/s12098-010-0006-4 20135267

[B11] Habib HM Mosaad YM Youssef HM Anti-cyclic citrullinated peptide antibodies in patients with juvenile idiopathic arthritis Immunol Invest 2008 37 849 857 10.1080/08820130802438057 18991100

[B12] Hromadnikova I Stechova K Pavla V Hridelova D Houbova B Voslarova S Anti-cyclic citrullinated peptide antibodies in patients with juvenile idiopathic arthritis Autoimmunity 2002 35 397 401 10.1080/0891693021000014970 12568120

[B13] Kasapcopur O Altun S Aslan M Karaarslan S Kamburoglu-Goksel A Saribas S Diagnostic accuracy of anti-cyclic citrullinated peptide antibodies in juvenile idiopathic arthritis Ann Rheum Dis 2004 63 1687 1689 10.1136/ard.2003.019331 15547097 PMC1754844

[B14] Kuna AT Lamot L Miler M Harjacek M Simundic AM Vrkic N Antibodies to mutated citrullinated vimentin and antibodies to cyclic citrullinated peptides in juvenile idiopathic arthritis Clin Chem Lab Med 2009 47 1525 1530 19842993 10.1515/CCLM.2009.288

[B15] Kwok JS Hui KH Lee TL Wong W Lau YL Wong RW Anti-cyclic citrullinated peptide: diagnostic and prognostic values in juvenile idiopathic arthritis and rheumatoid arthritis in a Chinese population Scand J Rheumatol 2005 34 359 366 10.1080/03009740510026634 16234183

[B16] Low JM Chauhan AK Kietz DA Daud U Pepmueller PH Moore TL Determination of anti-cyclic citrullinated peptide antibodies in the sera of patients with juvenile idiopathic arthritis J Rheumatol 2004 31 1829 1833 15338508

[B17] Ozawa R Inaba Y Mori M Hara R Kikuchi M Higuchi R Definitive differences in laboratory and radiological characteristics between two subtypes of juvenile idiopathic arthritis: systemic arthritis and polyarthritis Mod Rheumatol 2011 22 558 564 21984130 10.1007/s10165-011-0540-6

[B18] Syed RH Gilliam BE Moore TL Prevalence and significance of isotypes of anti-cyclic citrullinated peptide antibodies in juvenile idiopathic arthritis Ann Rheum Dis 2008 67 1049 1051 18556446 10.1136/ard.2007.084855

[B19] van Rossum M van Soesbergen R de Kort S ten Cate R Zwinderman AH de Jong B Anti-cyclic citrullinated peptide (anti-CCP) antibodies in children with juvenile idiopathic arthritis J Rheumatol 2003 30 825 828 12672206

[B20] Morbach H Dannecker H Kerkau T Girschick HJ Prevalence of antibodies against mutated citrullinated vimentin and cyclic citrullinated peptide in children with juvenile idiopathic arthritis Clin Exp Rheumatol 2010 28 800 20822716

[B21] Rantapaa-Dahlqvist S de Jong BA Berglin E Hallmans G Wadell G Stenlund H Antibodies against cyclic citrullinated peptide and IgA rheumatoid factor predict the development of rheumatoid arthritis Arthritis Rheum 2003 48 2741 2749 10.1002/art.11223 14558078

[B22] Plenge RM Recent progress in rheumatoid arthritis genetics: one step towards improved patient care Curr Opin Rheumatol 2009 21 262 271 10.1097/BOR.0b013e32832a2e2d 19365266

[B23] Klareskog L Stolt P Lundberg K Kallberg H Bengtsson C Grunewald J A new model for an etiology of rheumatoid arthritis: smoking may trigger HLA-DR (shared epitope)-restricted immune reactions to autoantigens modified by citrullination Arthritis Rheum 2006 54 38 46 10.1002/art.21575 16385494

[B24] Havemose-Poulsen A Westergaard J Stoltze K Skjodt H Danneskiold-Samsoe B Locht H Periodontal and hematological characteristics associated with aggressive periodontitis, juvenile idiopathic arthritis, and rheumatoid arthritis J Periodontol 2006 77 280 288 10.1902/jop.2006.050051 16460255

[B25] Lundberg K Kinloch A Fisher BA Wegner N Wait R Charles P Antibodies to citrullinated alpha-enolase peptide 1 are specific for rheumatoid arthritis and cross-react with bacterial enolase Arthritis Rheum 2008 58 3009 3019 10.1002/art.23936 18821669

[B26] Lundberg K Wegner N Yucel-Lindberg T Venables PJ Periodontitis in RA-the citrullinated enolase connection Nat Rev Rheumatol 2010 6 727 730 10.1038/nrrheum.2010.139 20820197

[B27] Toussirot E Roudier J Pathophysiological links between rheumatoid arthritis and the Epstein-Barr virus: an update Joint Bone Spine 2007 74 418 426 10.1016/j.jbspin.2007.05.001 17625943

[B28] Prahalad S Thompson SD Conneely KN Jiang Y Leong T Prozonic J Hierarchy of risk of childhood-onset rheumatoid arthritis conferred by HLA-DRB1 alleles encoding the shared epitope Arthritis Rheum 2012 64 925 930 21953520 10.1002/art.33376 PMC3276774

[B29] Hassfeld W Steiner G Hartmuth K Kolarz G Scherak O Graninger W Demonstration of a new antinuclear antibody (anti-RA33) that is highly specific for rheumatoid arthritis Arthritis Rheum 1989 32 1515 1520 10.1002/anr.1780321204 2597207

[B30] Nesher G Wilson VK Moore TL Osborn TG Hanna VE Antiperinuclear and anti-RA33 antibodies in juvenile chronic arthritis Ann Rheum Dis 1994 53 282 283 8203963 10.1136/ard.53.4.282-a PMC1005313

[B31] Tomoum HY Mostafa GA El-Shahat EM Autoantibody to heterogeneous nuclear ribonucleoprotein-A2 (RA33) in juvenile idiopathic arthritis: clinical significance Pediatr Int 2009 51 188 192 10.1111/j.1442-200X.2008.02667.x 19405913

